# Using motivational interviewing to facilitate death talk in end-of-life care: an ethical analysis

**DOI:** 10.1186/s12904-018-0305-5

**Published:** 2018-03-21

**Authors:** Isra Black, Ásgeir Rúnar Helgason

**Affiliations:** 1 York Law School, The University of York, York, UK; 2 Department of Public Health Sciences, Karolinska Institutet, Stockholm, Sweden; 3 Reykjavik University, Reykjavik, Iceland

**Keywords:** Palliative care, End-of-life, Motivational interviewing, Medical ethics, Death talk, Bereavement, Disclosure

## Abstract

**Background:**

Morbidity arising from unprepared bereavement is a problem that affects close personal relations of individuals at the end-of-life. The bereavement studies literature demonstrates that a lack of preparedness for a loved one’s death is a risk factor for secondary psychological morbidity among survivors. Short awareness time of death negatively correlates to preparedness for bereavement. The absence of disclosure of end-of-life diagnosis and prognosis to close personal relations (‘death talk’) between patients and loved ones, or health professionals and loved ones, may contribute to short awareness time of death. To increase awareness time of death, we might attempt to increase patient first-personal disclosure of end-of-life diagnosis and prognosis to loved-ones, and/or patient consent to health professional disclosure of the same.

**Main text:**

Interventions based on motivational interviewing in end-of-life care whose aim is to facilitate death talk, either by the patient directly, or by a health professional with the patient’s consent, may offer a part solution to the problem of unprepared bereavement. This paper evaluates the ethical permissibility of such interventions. We consider two ethical objections to using motivational interviewing in this way: first, that it is inappropriate for practitioners to seek disclosure as an outcome in this setting; second, that aiming at disclosure risks manipulating individuals into death talk.

While it need not be impermissible to direct individuals toward disclosure of end-of-life diagnosis/prognosis, the objection from manipulation implies that it is pro tanto ethically preferable to use motivational interviewing in a non-directive mode in death talk conversations. However, insofar as non-directive motivational interviewing requires more advanced skills, and thus may be more difficult to learn and to practise, we advance that it may be ethically permissible, all things considered, to employ directional, or specific outcome-oriented, motivational interviewing.

**Conclusion:**

Motivational interviewing interventions in end-of-life care whose aim is to facilitate death talk, either by the patient directly, or by a health professional with the patient’s consent may be ethically permissible, all things considered.

## Background

This paper evaluates the ethical permissibility of interventions based on motivational interviewing (henceforth MI) in end-of-life care whose aim is to facilitate ‘death talk’, that is, disclosure of end-of-life diagnosis and prognosis to close personal relations, either by the patient directly, or by a health professional with the patient’s consent. By increasing the incidence of death talk, it may be possible to reduce the incidence of unprepared bereavement, which may cause psychological morbidity among survivors. We argue that directional, or specific outcome-oriented, MI in this setting may be permissible, all things considered.

Unprepared bereavement has considerable health implications for survivors. The bereavement studies literature demonstrates that a lack of preparedness for a loved one’s death is a risk factor for secondary psychological morbidity among survivors. In an interview study of 122 individuals, Barry et al. found an association between survivors’ self-perceived lack of preparedness and complicated grief syndrome at baseline and follow-up, and major depressive disorder at follow-up respectively [1]. In a study of 222 bereaved carers of dementia patients, Hebert et al. found higher prevalence of depression, complicated grief, and anxiety among survivors who reported being unprepared for the patient’s death [2]. A Swedish population-based study by Hauksdóttir et al. of 691 widowers aged 38–61 whose wife had fatal cancer found an association between low preparedness for bereavement and anxiety, worrying, emotional numbness, little or no grief resolution, and sleep disorders four to five years post-loss [3]. Drawing on the same data set, Asgeirsdóttir et al. found, inter alia, an association between low bereavement preparedness and self-reported chronic pain among widowers aged 38–61 [4]. In another Swedish population-based study of 379 widows whose husband had fatal prostate cancer, Valdimarsdottír et al. found an association between short awareness time of death (< 24 h) and anxiety-related morbidity 2–4 years post loss [5].

The causes of bereavement unpreparedness are not well-known. Valdimirsdóttir et al. found that two potential predictors of short awareness time are communication between health professionals and (soon to be) survivors, and access to psychosocial support [5]. To the extent that short awareness time of death negatively correlates to preparedness for bereavement, it seems plausible that the aforementioned predictors also influence the incidence of secondary morbidity among the bereaved [6]. With regard to professional communication, it appears that non-disclosure of diagnosis and prognosis to patients, [7–10] as well as caregivers, [10–12] remains prevalent in professional practice. Health professionals may withhold end-of-life information for a variety of reasons, including a ‘fear of creating or worsening patient or caregiver distress’ [13]. This is notwithstanding little evidence to suggest that disclosure of diagnosis or prognosis is harmful to patients or caregivers [8, 9]. If patients at the end-of-life are unaware of their own short life expectancy, it follows that they cannot initiate conversations with loved-ones about death, or consent to disclosure by health professionals. In consequence, loved ones may be unprepared for bereavement. In addition, disclosure of diagnosis/prognosis to loved-ones by health professionals may be too proximate to the patient’s death to enable preparation for bereavement [12].

Even when health professionals provide adequate end-of-life information to the patient, loved-ones may be unable to prepare for bereavement if the patient abstains from first-personal disclosure of diagnosis/prognosis and withholds consent to professional disclosure of the same. As Skulason et al. observe, disclosure to close personal relations without the patient’s consent is unlawful in many jurisdictions [14]. Moreover, unconsented disclosure by a health professional may trigger regulatory proceedings for violation of patient confidentiality.^1^

In order to reduce secondary psychological morbidity among the bereaved, therefore, we might attempt to increase patient first-personal disclosure of end-of-life diagnosis and prognosis to loved-ones, and/or patient consent to health professional disclosure of the same. To this end, interventions based on motivational interviewing may appear promising.

## Main text

In this section, we explain what MI is and how an MI-based intervention might facilitate death talk. Second, we consider whether using MI in this way is ethically permissible.

### A part solution? Motivational interviewing in end-of-life care

In this sub-section, we outline how an MI-based intervention might facilitate increased disclosure of terminal diagnosis and prognosis to loved-ones. First, however, it is necessary to say a little about MI and its evidence base.

#### What is MI?

MI is:

A collaborative, goal-oriented style of communication with particular attention to the language of change. It is designed to strengthen personal motivation for and commitment to a specific goal [or target behaviour] by eliciting and exploring the person’s own reasons for change within an atmosphere of acceptance and compassion [15].

Two components of MI are thought to be active in influencing behaviour: a relational component; and a technical component [15].^2^

The relational, person-centred ‘spirit’ of MI, comprises four interrelated dispositions [15]. First, the MI spirit is one of *partnership*. As Miller and Rollnick argue, MI is an ‘active collaboration between experts’ in which the ‘interviewer seeks to create a positive interpersonal atmosphere that is conducive to change but not coercive’ [15]. Second, the MI spirit involves *acceptance*, which in turn comprises four elements: recognition of the interviewee’s ‘*absolute worth*’; *empathy*, conceived as ‘an active interest in and effort to understand the other’s internal perspective’; respect for *autonomy*, that is, recognition that the final decision with regard to behaviour change rests with the interviewee; and *affirmation*, the practice of seeking and acknowledging the interviewee’s strengths and efforts [15]. Third, the MI spirit requires *compassion*, insofar as interviewers must commit ‘to pursue the welfare and best interests of the other’. Fourth, the MI spirit calls on practitioners to *evoke* the client’s own motivation and resources with regard to behaviour change [15].

The technical component of MI involves four overlapping processes that are ‘both sequential and recursive’ [15]. *Engagement* is ‘a process by which both parties establish a helpful connection and working relationship’ [15]. *Focusing* cultivates a particular conversational direction or target [15]. The object of focusing might be substantive, for example, ‘drinking less’, or instrumental, for example, ‘choosing between treatment options’. Once focus is established, the interviewer progresses to *evocation* through ‘selectively eliciting and reinforcing [*change talk*—]the client’s own arguments and motivations for change’, while being mindful not to evoke *sustain talk* favouring the behavioural status quo [16]. Focusing and evocation in particular contribute to MI’s distinctiveness, since unlike ‘traditional conceptions of client-centred counselling[… MI is] consciously goal-oriented, in having intentional direction toward change’ [16]. Finally, once the interviewee has reached a sufficient level of readiness for change, a *planning* process seeks to develop commitment to change and formulate a change plan [15].

A number of core skills operationalise the spirit and method of MI: open questions, affirmation, reflections, and summaries [15, 17]. *Open questions* invite reflection and elaboration on the part of the interviewee, and promote a collaborative relationship between the parties [15]. *Affirmation* asks the interviewer to adopt the mindset of ‘accentuate the positive’, which is instantiated by acknowledgement of positive dispositions on the part of the interviewee [15]. *Reflections* attempt to deepen the interviewer’s understanding by selectively clarifying the interviewee’s meaning, and offering the latter an opportunity to listen back to her thoughts and feelings [15]. *Summaries* are reflections that collate the interviewee’s utterances. Summaries may serve to establish alliance, tie together material, transition between the stages of MI, and provide an opportunity for the interviewee to add material that furthers the interviewer’s understanding [15].

#### The evidence for MI

MI has a robust evidence base in clinical trials across a wide range of applications [18–22]. This evidence demonstrates that MI is ‘effective both in reducing maladaptive behaviors[…] and in promoting adaptive health behavior change[…] for use when client ambivalence and motivation appear to be obstacles to change’ [23]. The trial evidence also shows that MI is efficacious in small doses—in brief consultations of 15 min of less, and in interventions of one to four sessions [15]. MI has been widely disseminated in practice; however, its effectiveness in real-life settings is yet to be established with rigour [24, 25].

#### Facilitating disclosure through MI in end-of-life care

The development of MI-based interventions for end-of-life care is perhaps unsurprising. Pollak et al. observe that resistance and ambivalence is common in palliative care conversations between professionals and patients and/or loved-ones, for example, opposition to proposed treatment plans, and uncertainty about which option to take when all available are non-ideal [17]. Since MI is designed to help individuals address ambivalence, it may be an appropriate clinical tool when ambivalence is present in end-of-life settings. Moreover, the spirit and method of MI are broadly synergetic with the ethos of palliative care, insofar as both emphasise ‘listening to patients, understanding patients’ motivations and values, and empowering patients’ [17].

MI-based interventions in palliative care may be directional or non-directive. In the former case, the interviewer seeks a substantive outcome. For example, Skulason et al. detail an MI-based evocation intervention designed to facilitate patient engagement in death talk with a hospital chaplain [14]. In the latter case, no substantive outcome is sought, although an instrumental aim may exist, that is, for the patient to exercise her autonomy through choosing between treatment options, planning for future incapacity, expressing preferences etc. For example, Ko et al. have piloted a staged MI-based intervention aimed at enhancing advance care planning that evokes motivation to plan for the end-of-life among interviewees, but remains neutral as to the content of the interviewee’s plans [26]. Pollak et al. have developed a non-directional MI-based protocol for palliative care conversations, which is yet to be trialled in a clinical setting [27]. We briefly outline how directional and non-directive approaches to death talk might work below.

As a starting point, individuals may be ambivalent about disclosure of end-of-life diagnosis/prognosis to loved-ones. For example: on the one hand, an individual may wish to disclose so that loved-ones can prepare for bereavement, or in order to have support while dying, itself essential to good palliative care; on the other hand, an individual may wish not to disclose out of a desire to maintain hope of recovery, or to spare loved-ones trauma (ambivalence need not be based on true beliefs), or because of estrangement or other complicating interpersonal factors.

Health professionals might employ MI to help patients resolve ambivalence about engaging in death talk by, within an environment consistent with the spirit of MI, eliciting and reinforcing statements that point toward disclosure to loved-ones, while avoiding dwelling on utterances that count against it. If MI is successful, and disclosure occurs first personally or via a professional, it may reduce the incidence of psychological morbidity among the bereaved by facilitating greater preparedness for the death of a loved one.

By contrast, a non-directive MI-based intervention would not seek disclosure of end-of-life diagnosis/prognosis to loved-ones, although it might take a decision about death talk as its target behaviour. Thus rather than selective evocation of reasons to disclose, the health professional would adopt a neutral position (*equipoise*), and ‘explore thoroughly both the pros and the cons [of disclosure…] in a balanced way’ [15].^3^

The idea, therefore, would be to consider the individual’s ambivalence against a backdrop of her values, in order to enable her to make a value-consistent decision, or at least better understand her ambivalence. Of course, a non-directive approach would not necessarily increase the incidence of death talk. Indeed, Miller and Rose submit that ‘[t]here are both theoretical and empirical reasons to expect that equally exploring the cons and pros of change with ambivalent clients would impede rather than promote change’ [28].

### The ethics of MI at the end-of-life

Would it be ethically permissible to take a directional MI approach toward disclosure of end-of-life diagnosis/prognosis? In this sub-section, we discuss two potential objections to such an approach: inappropriate target behaviour and manipulation. We also consider an objection to non-directive MI.

#### Inappropriate target behaviour

Pollak et al. argue that there is a ‘fundamental difference’ between MI and palliative care communication, in that many applications of MI, for example, smoking cessation, are directional (they aim at a specific target behaviour), whereas:

In palliative care consultations, there is no objectively correct answer about what constitutes a good death for the patient. Therefore, MI cannot be directive when used in palliative medicine. The skilled palliative care clinician[…] elicits the patient’s values and seeks to assist patients or proxies in making autonomous choices that will be consistent with those values [17].

Pollak et al. couch a normative claim about what a palliative care professional *ought* to do as a descriptive claim about what she *is able* to do. Clearly it would be feasible to enter an end-of-life conversation with a goal in mind, for example, agreement to withdraw life prolonging treatment. Rather, Pollak et al’s. argument is that it would not be *appropriate* to seek a specific outcome in this setting, in virtue of uncertainty about what is good for the patient. If Pollak et al’s. thesis holds, by analogy we ought not to use MI to facilitate death talk; rather, to the extent that any purposeful approach is warranted, non-directive (or *instrumental*) MI is the appropriate stance to adopt.

To specify in greater detail, the argument, mutatis mutandis, seems to be that only non-directive MI, that is, an MI-based intervention in which the interviewer seeks only that the patient make a decision whether to engage in death talk with loved ones, is morally permissible. Thus the interviewer ought not to invest either of the outcomes {disclosure, non-disclosure} available to the patient and should not, in consequence, utilise MI in order to facilitate disclosure. However, if non-directive (instrumental) MI is permissible, it follows that it is permissible, within a person-centred environment, to employ the MI processes and to utilise the MI skills in order to focus on and evoke talk that favours deciding whether to disclose, while taking care not to elicit talk that eschews taking a decision. In addition, it is permissible to engage the MI processes and skills in order to explore death talk, although crucially the interviewer would be ethically required to employ the evocation process bi-directionally, that is, to elicit the reasons for and against disclosure, without selectively reinforcing either set. Of course, the claim that using a directional MI-based intervention in this setting is ethically impermissible requires interrogation.

We are unconvinced that there is never an objective answer to what constitutes a bad death for the patient. For example, there may be circumstances in which an individual’s wishes for her own death profoundly conflict with her best interests, for example, when she requests the application of life prolonging measures at any cost [29]. Death under these conditions seems plausibly very bad from a prudential standpoint. Consequently, it may be permissible for end-of-life care professionals to direct against ‘extreme’ patient wishes. However, more often there will be interventions on the menu whose benefit to the patient depends on the latter’s preferences. Consider, for example, the choice of {sedation, more comfort} or {lucidity, less comfort}. In such circumstances, it may indeed be inappropriate to take a stance on which outcome is best, because of the connection between a patient’s wishes and preferences and her best interests.

With regard to death talk, it seems plausible that the prudential goodness of disclosure depends on the patient’s wishes and preferences and her situation. The patient may have good reasons not to disclose, for example, family estrangement, that make it difficult to determine that disclosure promotes her welfare. We might accept, therefore, that using MI to facilitate death talk is inappropriate for the general reasons Pollak et al. give.

Of course, it is possible to argue that disclosure is objectively good not because it is necessarily good for the patient, but because of the benefits that accrue to loved ones in being able to prepare for bereavement. However, if we know that in some cases disclosure would not be good for the patient, we must to defend a view of interpersonal aggregation that discounts her ill-being or that holds it secondary to the prudential interests of others. Such a view seems out of step with the standard conception of medical ethics, which sets the patient as the primary unit of ethical concern [30].

It is possible, however, for proponents of using MI to facilitate death talk to sidestep the preceding concerns, and thereby argue that it is permissible to have disclosure as a target behaviour. This is because, if MI works according to its theoretical causal model, an intervention aimed at facilitating death talk will not work for patients who deep down do not favour disclosure. Black and Forsberg write that ‘MI’s causal role in behaviour change consists in highlighting the contrast between status quo behaviour(s) and deeply held values and beliefs’ [31]. Therefore, as Miller and Rollnick argue, ‘[u]nless the change is in some way consistent with the client’s own goals or values, there is no basis for MI to work’ [15]. The argument is that we can facilitate death talk without ethical anxiety that directing the conversation toward that outcome might not be good for the patient, or might require endorsement of a novel theory of medical ethics; the patient will choose disclosure only if it is best for her. However, it is necessary to address a further ethical concern about manipulation, which stems from a worry about whether MI works as theorised.

#### Manipulation

Wilkinson argues that manipulation consists in ‘[intentional conduct that] infringes upon the autonomy of the victim by subverting and insulting their decision-making powers’ [32]. In what way might MI be manipulative? Black and Forsberg suggest that the selective reinforcement of *any* utterances, not just those which align with core values and beliefs, may influence behaviour [31]. The idea is that the evocation of talk that favours a distinct outcome may distort or pervert the interviewee’s decision-making processes by minimising potentially cogent reasons against that choice. In so doing, MI potentially inhibits the ability of the interviewee to reach an adequately deliberated decision.

Against the manipulation objection, the evidence that MI is not 100% effective even when delivered by proficient counsellors lends support to the claim that MI operates (as theorised) on intrinsic motivation [33]. However, it cannot confirm it; it does not follow from the fact that some people were not victims of manipulation that no one was manipulated. Moreover, Black and Forsberg observe that it may be challenging to establish whether MI is manipulative in any given case without sufficient prior knowledge of an individual’s values and beliefs prior to an MI intervention [31]. Furthermore, we have no representative data in respect of individuals’ preferences about death talk, and as such we cannot estimate the risk of MI manipulating patients into death talk [31].

In consequence, it may be that non-directive (instrumental) MI is pro tanto the appropriate approach to conversations about death talk. However, the difficulty of maintaining substantive neutrality may mean that MI is the ethical approach all things considered.

#### The difficulty of non-directive MI

Substantial training in MI is required to develop the skills that may enable practitioners to influence interviewee behaviour [24, 34–36]. For example, in a recent systematic review of MI dissemination in the substance use disorder treatment field, Hall et al. observe that ‘for many practitioners, achieving proficiency in MI may take years’ [24]. Even if we are more optimistic about the nature and quantity of training in MI required for sustained competent practice, MI dissemination in end-of-life care is likely to be challenging and may require systemic change [37].^4^

However, the difficulty of learning MI should be of significant concern also to advocates of non-directive MI-based interventions, to the extent that maintaining substantive neutrality may necessitate a yet more advanced set of MI skills. In an earlier edition of *Motivational Interviewing*, Miller and Rollnick concede that non-directive counselling may require ‘a still higher level of clinical skilfulness than the directive variety of counseling, because one must avoid inadvertently tipping the scales in one direction or the other’ [38]. To explicate this claim, recall that non-directive MI in the death talk setting requires attentiveness to an even balance of the reasons that count in favour and against disclosure of diagnosis/prognosis; the interviewer must maintain equipoise. Metaphorically-speaking, non-directive MI is a tightrope walk; precise balance is required if the activity is to succeed. By contrast, in a directional MI-based death talk intervention, the interviewer should selectively elicit and reinforce the patient’s reasons for disclosure, and avoid evoking talk that favours non-disclosure. Figuratively, we might imagine a set of stepping stones across a shallow stream; a misstep may result in wet feet, but need not prevent crossing. Just as the tightrope seems plausibly more difficult that the stepping stones, so too non-directive MI may be more challenging than directional MI. As Black and Forsberg argue, *mutatits mutandis*, it may be ‘unrealistic, therefore, to think that [interviewers] would be able to use [non-directive MI]’ in conversations about death talk [31].

Once we factor in the practicalities of learning MI, it may be ethically permissible, all things considered, to implement a directional MI-based approach to death talk conversations, subject to the requirement that the interviewer is upfront about her aims and methods, and gains the interviewee’s consent to the intervention. We are not certain that under these circumstances consent would render any manipulation permissible, [32] but we would avoid the situation in which non-directive MI were promised but directional counselling delivered—itself an autonomy violation.

## Conclusion

In this paper, we identified morbidity arising from unprepared bereavement as a problem that affects close personal relations of individuals at the end-of-life. We suggested that the absence of death talk between patients and loved ones, or health professionals and loved ones, is a contributing factor to unprepared bereavement. We argued that a Motivational Interviewing intervention aimed at facilitating death talk may offer a part solution to the problem of unprepared bereavement. Subsequently, we considered two ethical objections to using MI in this way. We argued that while it need not be impermissible to direct toward disclosure of end-of-life diagnosis/prognosis, the objection from manipulation implies that it is pro tanto ethically preferable to use non-directive MI counselling in death talk conversations. However, insofar as non-directive MI may be more difficult to learn and practise than directional MI, we advance that it may be ethically permissible, all things considered, to have disclosure as the target behaviour of an MI-based death talk intervention.

Ultimately, there is a fine balance in respect of whether it is best, ethically-speaking, to use directional or non-directive MI to respond to ambivalence about disclosure of end-of-life diagnosis/prognosis. Regardless of which intervention is best, we submit that both are ethically advantageous compared to unsystematic approaches, not least because both interventions will facilitate conversations about disclosure between physicians and patients, and thereby increase the likelihood that patients themselves will have a good death.

## Abbreviations

MI: Motivational interviewing

## Footnotes

1 This is implicit in eg [39];

2 A model that describes the causal processes of MI in respect of behaviour change is yet to be validated empirically. However, partial empirical support for MI’s constituent elements can be found in [40–42]. See [23] for a hypothetical causal model;

3 It should be clear that directive MI and non-directive (instrumental) MI share a common skill base. Moreover, this foundation is shared with entirely non-directive, or ‘decisional balance’ counselling, [43] which Miller and Rollnick differentiate from MI as a ‘contrasting [approach] for responding to ambivalence’ [15]. Unlike the variants of MI discussed, the decisional balance practitioner actively avoids *any* attempt to influence ‘the client’s movement in any particular direction’ [28].

4 Two specific challenges in respect of MI implementation relate to uncertainty about the level of interviewer proficiency that is sufficient to influence interviewee behaviour, and the kind of training that is likely to produce proficient MI practitioners [37]. A related concern speaks to the essential, but difficult, task of assessing fidelity to MI, and measuring the impact of MI on subsequent interviewee behaviour [24].
